# Dementia Worry and Depression: A Systematic Review

**DOI:** 10.1093/geroni/igaf122.456

**Published:** 2025-12-31

**Authors:** Sabine Lohmar

**Affiliations:** West Virginia University, Morgantown, West Virginia, United States

## Abstract

Dementia worry (DW), a response ranging from mild concern to severe anxiety about developing dementia, has been increasingly recognized as a significant psychological concern (Kessler et al., 2012). It is unclear, though, whether DW is associated with psychosocial variables such as depression (e.g., Kinzer & Suhr, 2016; Spalding et al., 2024) and no systematic reviews exist on the topic. Investigating this relationship is crucial to understanding how DW develops and is maintained. This systematic review evaluates and synthesizes the current literature on the relationship between DW and depressive symptoms. A comprehensive search of five databases was conducted between September and October of 2024. Studies were included if they were peer-reviewed, available in English, and statistically examined the relation between DW and depressive symptoms. This process identified 22 studies with 19 distinct samples conducted in six countries, encompassing 8,581 total participants. Data on sample characteristics, measures, statistical analyses, and results were extracted and compiled. Each study’s quality was assessed with the Appraisal Tool for Cross-Sectional Studies (AXIS). Conclusions were drawn while weighing the strengths and weaknesses of each study. Results of this review suggest that DW and depressive symptoms are positively related, but that this relationship may vary in strength based on exposure to dementia, gender, and strength of DW. Moreover, this association may be related by avoidant behaviors, potentially exacerbating distress and delaying cognitive testing. Future research should investigate how exposure to dementia influences the strength of the relationship between DW and depression.

